# Supplementary material for: Risk factors and economic burden of postoperative anastomotic leakage related events in patients who underwent surgeries for colorectal cancer
Source: PLoS One. 2022 May 18;17(5):e0267950. doi: 10.1371/journal.pone.0267950 (PMC9116683; doi:10.1371/journal.pone.0267950)
Supplement: S1 Table — (DOCX) [file pone.0267950.s001.docx]

**S1 Table. Economic Outcomes of the Presence of Additional Intervention Cases(AIC)**

|  | With AIC | | Without AIC | | Total | | P-value |
| --- | --- | --- | --- | --- | --- | --- | --- |
|  | (N=5,483) | | (N=114,762) | | (N=120,245) | |  |
| Total costs for the index hospitalization, (USD) |  |  |  |  |  |  | <.0001 |
| Mean ± SD | 9,368 | ± 7,053 | 7,163 | ± 3,617 | 7,263 | ± 3,868 |  |
| Median | 7,585 | | 6,466 | | 6,508 | |  |
| Q1, Q3 | 5,831 | - 10,406 | 5,219 | - 8,113 | 5,239 | - 8,186 |  |
| Total costs for the period from index date to last f/u date, (USD) |  |  |  |  |  |  | <.0001 |
| Mean ± SD | 11,391 | ± 7,638 | 7,562 | ± 3,668 | 7,737 | ± 4,018 |  |
| Median | 9,420 | | 6,878 | | 6,950 | |  |
| Q1, Q3 | 7,134 | - 13,106 | 5,553 | - 8,604 | 5,592 | - 8,743 |  |
| Total costs for the readmission, (USD) |  |  |  |  |  |  | <.0001 |
| N | 4,193 | | 52,829 | | 57,022 | |  |
| Mean ± SD | 3,481 | ± 4,250 | 1,376 | ± 1,155 | 1,643 | ± 1,987 |  |
| Median | 2,093 | | 1,166 | | 1,241 | |  |
| Q1, Q3 | 1,105 | - 4,201 | 671 | - 1,744 | 705 | - 1,866 |  |
| Los for the index hospitalization, (duration) |  |  |  |  |  |  | <.0001 |
| Mean ± SD | 17.41 | ± 13.99 | 14.23 | ± 8.39 | 14.37 | ± 8.75 |  |
| Median | 13.00 | | 12.00 | | 12.00 | |  |
| Q1, Q3 | 10.00 | , 20.00 | 10.00 | , 16.00 | 10.00 | , 16.00 |  |
| Los for the readmission, (duration) |  |  |  |  |  |  | <.0001 |
| N | 4,193 | | 52,829 | | 57,022 | |  |
| Mean ± SD | 20.59 | ± 16.79 | 14.15 | ± 10.86 | 14.63 | ± 11.53 |  |
| Median | 16.00 | | 13.00 | | 13.00 | |  |
| Q1, Q3 | 9.00 | , 27.00 | 7.00 | , 18.00 | 7.00 | , 18.00 |  |

1USD=1,150 Korean won; USD, U.S. dollar

AL=anastomotic leakage; SD=standard deviation
